# Data-mining Techniques for Image-based Plant Phenotypic Traits Identification and Classification

**DOI:** 10.1038/s41598-019-55609-6

**Published:** 2019-12-20

**Authors:** Md. Matiur Rahaman, Md. Asif Ahsan, Ming Chen

**Affiliations:** 1 Department of Bioinformatics, College of Life Sciences, Zhejiang University, Hangzhou, 310058 China; 2 Department of Statistics, Faculty of Science, Bangabandhu Sheikh Mujibur Rahman Science & Technology University, Gopalganj, 8100 Bangladesh

**Keywords:** Plant sciences, Statistics

## Abstract

Statistical data-mining (DM) and machine learning (ML) are promising tools to assist in the analysis of complex dataset. In recent decades, in the precision of agricultural development, plant phenomics study is crucial for high-throughput phenotyping of local crop cultivars. Therefore, integrated or a new analytical approach is needed to deal with these phenomics data. We proposed a statistical framework for the analysis of phenomics data by integrating DM and ML methods. The most popular supervised ML methods; Linear Discriminant Analysis (LDA), Random Forest (RF), Support Vector Machine with linear (SVM*-l*) and radial basis (SVM-*r*) kernel are used for classification/prediction plant status (stress/non-stress) to validate our proposed approach. Several simulated and real plant phenotype datasets were analyzed. The results described the significant contribution of the features (selected by our proposed approach) throughout the analysis. In this study, we showed that the proposed approach removed phenotype data analysis complexity, reduced computational time of ML algorithms, and increased prediction accuracy.

## Introduction

Phenomics technologies have been rapidly developed in plant science. They provide a great potential to gain more valuable information than traditionally destructive methods of plant phenotyping. It carried out large-scale plant phenotyping facilities that acquire a large number of images of hundreds of plants simultaneously. With the aid of automated image processing, the phenotype-image data are converted into phenotype-feature matrices^1^. It is a great challenge to find a suitable techniques or methodologies to analysis phenotype data in the context of high-throughput phenotyping. However, extracting data patterns, data assimilation, and features (traits) identification from this large corpus of data requires the use of data mining (DM) and machine learning (ML) tools^1–3^. Supervised and unsupervised DM and ML algorithms are promising tools to assist in the analysis of complex data sets; novel approaches are needed to apply them on phenotyping data of mature plants^4^.

In agricultural development, there is a demand to control diseases and numerous stresses to maintain food quality worldwide and to reduce food-borne illness originated from infected plants. A wide variety of plant stresses and diseases caused by the environmental factors, for example, light quantity, light quality, CO_2_, nutrients, air humidity, water, temperature, drought, salinity or other organisms such as fungi, bacteria, and viruses. They hinder agricultural development by disturbing grain production and quality through competing with these factors. Thus, it is important to detect and classify the plant infestations^5^.

Supervised ML methods are useful for biological and plant image analysis^1,4,6–9^. Linear Discriminant analysis (LDA) is a popular supervised ML method widely used for biomedical data classification^5,10,11^. Among the supervised ML algorithms, Random Forest (RF) is a non-parametric method has been applied in several biological fields for gene selection, protein sequence selection and disease prediction^12–14^. RF has been used for accurate prediction of plant biomass from image-based features^9^. Support Vector Machine (SVM) is another powerful supervised ML method which can be trained to classify individuals in high-dimensional space^15^. SVM has been widely used in the various biomedical fields as well as neuro-image classification, plant image classification, biomass prediction, stress plant identification based on image-derived features^9,16–19^. In most cases, symptoms of stress and disease in plants result are the change of the plant color^9,10^. ML approaches can be used to classify color-related traits, which obtain from the plant phenotype image pixels under the biotic and abiotic conditions^1^.

In high-throughput plant studies, most informative phenotypic traits offer better data analysis results. Plant biologists train classification model; however need to improve the training data by inspection of the significant phenotypic traits. Identifying candidate traits from ten to hundred or even more image-derived phenotypic traits for QTL (quantitative traits locus) or GWAS (genome-wide association study) study is also an important challenging research topic to bridge the genotype-phenotype gap^9^. This analysis is highly essential in resisting environmental stress rates in agronomic importance^20–22^. Traditional statistical methods are extensively used to deal with genomic data analysis^1^. A powerful statistical approach or analytical framework is essential for describing crop cultivars by integrating traditional or novel methods with the complex traits set^4^.

In this study, we propose a statistical framework for quantitative image data pre-processing, and improve the training dataset for estimating ML model by inspecting important phenotypic traits using DM technique. We explore how performance varies with the selected number of traits, and investigate the performance of each ML method (classifier) mentioned earlier. We used plant phenotype dataset that has different types of phenotypic features (geometrical and physiological). We also used cross-validation technique, which is important because it is needed to evaluate the performance of a classifier, and needs to be done many times in training a classifier in an iterative fashion. The next part describes the dataset, the approach and the supervised ML methods used in this study. The last part consists of results and discussions.

## Materials and Methods

### Data description

#### Simulated data

To investigate the performance of ML methods based on selected features through our proposed approach, we generated simulated training and test dataset from *m* = 2 (Π_1_ and Π_2_) multivariate normal distributions and the data structure is:

$$D:{\Pi }_{1}\sim {n}_{1}{N}_{p}({\mu }_{1},{V}_{1}),{\Pi }_{2}\sim {n}_{2}{N}_{p}({\mu }_{2},{V}_{2}).$$

Where *n*_1_ and *n*_2_ are the numbers of individuals; ${N}_{p}({\mu }_{1},{V}_{1})$ and ${N}_{p}({\mu }_{2},{V}_{2})$ are *p*-variate normal distributions with mean vector ***μ***_1_ and ***μ***_2_, and covariance matrix *V*_1_ and *V*_2_, respectively. We considered here, *V*_1_ = *V*_2_ = *V*; and ***μ***_2_ = ***μ***_1_ + ϵ with ϵ = 0, 1,…, 10 such that ***μ***_1_ = ***μ***_2_ for ϵ = 0, otherwise ***μ***_1_ ≠ ***μ***_2_, where the scalar quantity ϵ denotes the common difference between two corresponding mean components of ***μ***_1_ and ***μ***_2_. We considered constant covariance matrices for the normal populations and the generated data vectors are arranged in a *n* × *p* matrix to obtain training and test data sets respectively, where *n* = *n*_1_ + *n*_2_.

#### Plant phenomics data

The Leibniz Institute of Plant Genetics and Crop Plant Research (IPK), Gatersleben, Germany has generated a high-throughput phenomics dataset. We downloaded the quantitative phenomics dataset from http://iapg2p.sourceforge.net/modeling/#dataset, and the details description of this dataset is available at Chen *et al*.^9^. The summarized description of the dataset according to the Chen *et al.*^9^ as follows:

A mini core set of 16 German two-rowed spring barley cultivars and two parents of a DH-mapping population (cv Morex and cv Barke) were screened. Plants grew under controlled greenhouse conditions and were phenotyped using the automated LemnaTec-Scanalyzer 3D (LemnaTec GmbH, Aachen, Germany) phenotyping and imaging platform consisting of conveyor belts, a weighing and watering station, and imaging sensors. The experiments were performed under two treatments: well-watered (control treatment) and water limited (drought stress treatment). Drought stress was imposed by intercepting water supply from 27 days after sowing until days 44. Stressed plants were re-watered at days 45. Control plants remained well watered. After the stress period (27–44 days), all plants were watered to 90% field capacity (FC) and kept well-watered again until the end of the experiment. The greenhouse growth conditions were set to 18 °C and 16 °C during the day and night, respectively. The daylight period lasted ~13 h started at 7 AM. During each treatment, six plants per DH parent and nine plants per core set cultivar were tested. For each plant, top and side cameras were used to capture images daily at three different wavelength bands: visible light, FLUO, and NIR.

Chen *et al*.^9^ performed image analysis through IAP software to extract quantitative information from the barley plant images^23^. Images were exported and analyzed using the barley analysis pipeline with optimized parameters. Image processing operations included steps: pre-processing, to prepare the images for segmentation; segmentation, to divide the image into foreground and background parts of the images, and feature extraction. The analyzed features were exported in .csv file format.

### Phenomics data processing and features selection

**Figure 1:**
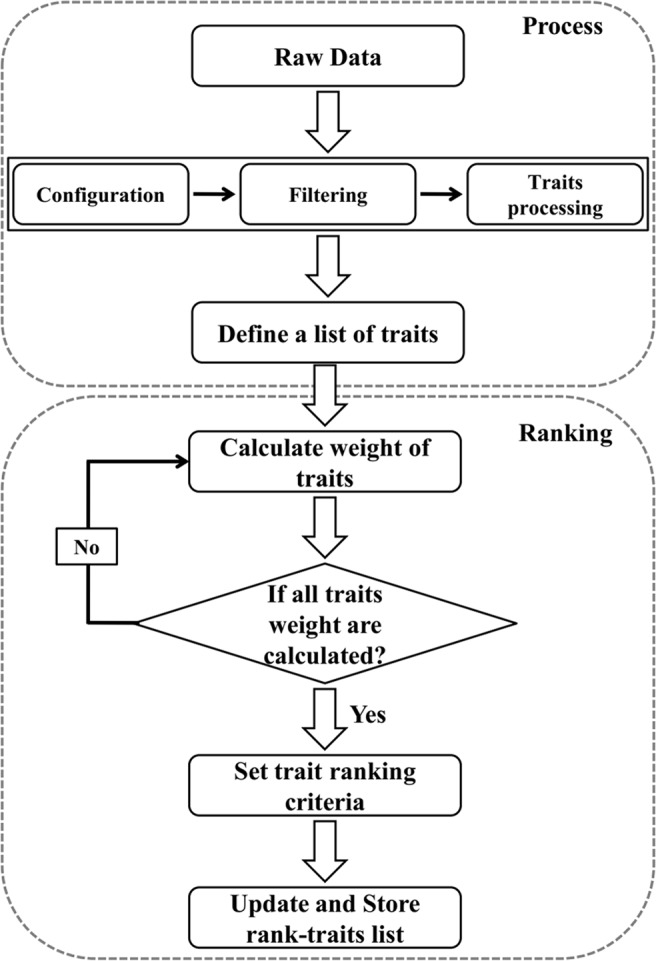
Framework of plant phenotype image-based traits (features) selection.

We proposed a statistical framework (Fig. 1) which is depicted in two phases: (a) Processing and (b) Ranking. A description of the framework elements are given below.

#### (a) Data pre-processing and features selection (Processing)

Given a set of phenotype data Ω_*n*_, we need to set data configuration based on color, shape structure, genotype, etc. for plotting and frequently used in the analysis. After that data filtering is needed, for example, removing ‘0’ values (in the image data are empty values), outlier detection, trait reproducibility assessment. For outlier detection, Grubbs test^24^ is a useful method based on assumption of the normal distribution of phenotype data points for repeated measures on replicated plants of a single genotype for each trait^9^. Bonferroni Outlier Test is another outlier detection method for identifying outliers from the image dataset, and need to remove outliers that could bias the results^25^. Then feature processing needs to continue, reasoned that phenotypic information should be more robust and informative. Features reproducibility test can be evaluated by the Pearson correlation coefficient. Resulting data sets may contain redundant features that are correlated with each other. To remove this problem and feature selection, stepwise variable selection using variance inflations factors^9^, principal component analysis^25^, RF^4^ are useful methods to get an optimal set of meaningful features.

#### (b) Features ranking by SVM-RFE (Ranking)

In this step, we have described phenotypic features ranking procedure using a ML method called Support Vector Machine-Recursive Feature Elimination (SVM-RFE). The SVM-RFE algorithm is an iterative procedure for SVM. A cost function β computed on training samples is used as an objective function. Expanding β in Taylor series to the second-order using the OBD algorithm^26^, and neglecting the first order-term at the optimum of β, yielding:

$$\Delta \beta (i)=\frac{1}{2}\frac{{\delta }^{2}\beta }{\delta {{w}_{i}}^{2}}(\Delta {{w}_{i}}^{2})$$

Here, ${{w}_{i}}^{2}$ was used as a ranking criterion^27,28^. We present below the outlines of the SVM-RFE for phenotype dataset as follows:

Features Ranking

**Table:** 

1. Procedure: Process (*Ω*, *K*)
Where Ω is phenotypic traits space, *K* is the set of labels (treatment or genotype)
2. Ψ_s_ ← Trait Selection (*Ω*, *K*)
3. Inputs: Training sample (Processed phenotypic image dataset)
$${X}_{0}={[{x}_{1\times {\varPsi }_{x}},{x}_{2\times {\varPsi }_{s}},\ldots ,{x}_{k\times {\varPsi }_{s}},\ldots ,{x}_{n\times {\varPsi }_{s}}]}^{T}$$
4. Group labels *K* = {0,1,…*m*}
5. Initialize: Ψ_s_ = [1, 2,…, *p*]; surviving traits
6. Trait ranked list, *r* = []; Repeat until ψ_s_ = []
7. α ← *svm*-train(*X*_0_, *K*); train the classifier.
8. w ← $\sum _{t}{\alpha }_{t}{X}_{t}{K}_{t}$; the weight of each selected trait of *t*-th training pattern.
9. *R*_*i*_ ← (*w*_*i*_)^2^, ∀ *i*; ranking criteria for the *i*-th trait.
10. g ← *argmin*(*R*); trait with the lowest ranking.
11. *r* ← [Ψ_s_*(g), r*]; renew the trait-ranking list.
12. Ψ_s_ ← Ψ_s_ (1:g-1, g + 1:length(ψ_s_)); eliminate the trait with lowest ranking.
13. return ()
14. End procedure.

### Supervised machine learning methods

Supervised learning have input variables (x) and an output variable (y) and we use an algorithm to learn the mapping function from the input to the output.

$${\rm{y}}={\rm{f}}({\rm{x}})$$

The goal is to approximate the mapping function. When we have new input data (x) that we can predict the output variables (y) for that data. It is called supervised learning because the process of an algorithm learning from the training dataset. The algorithm iteratively makes predictions on the basis of training data and learning stops when the algorithm achieves an acceptable level of performance.There is no single supervised ML (classification) algorithm which outperforms on all datasets. Every classification method has its own strengths and limitations^29,30^. From the literature review, in this study, we have tested popular three ML algorithms for classification: Linear Discriminant Analysis (LDA); Random Forest (RF); and Support Vector Machine (SVM). SVM we differentiated based on linear and radial basis kernel functions. These algorithms belong to the type of supervised classification require of a training stage before performing the classification process. The details of the implementation and tuning of the parameters of these classifiers are as follows:

- Linear Discriminant Analysis (LDA): Linear Discriminant Analysis is a useful ML algorithm when features are linearly independent and normally distributed. LDA tries to maximize the separation between classes by estimating class boundedness as a linear combination of the features. It does not need parameter tuning. We choose this supervised classifier because it is conventionally considered to be a good benchmark classifier^31^. R package *MASS* is used for LDA method.
- Random Forest (RF): Random forest is a classifier that consists of many decision trees. It outputs the class that is the mode of the classes output by individual trees. To achieve excellent performance, RF requires tuning parameter, *mtry*, the number of input features tried at each split for building each tree^4,12,32^. We used the *cforest* function in the R *Party* package and, *mtry* = *p* was tuned, where *p* is the amount of selected phenotypic features.
- Linear support vector machine (SVM-*l*): Linear support vector machine is used for large data sets where with/without nonlinear mapping gives similar performance^31,33^. To reduce training and testing times, SVM-*l* requires only one hyper parameter C. The search for the optimal hyper parameter C was performed on values C ∈ [2^0^, 2^1^, …, 2^4^].
- Support vector machine with radial basis function (SVM-*r*): Generally, the Support vector machine with radial basis function classifier is better in performance and is tolerant to irrelevant and interdependent features^31,33^. SVM-*r* is a useful method when data is not linearly separable but slower because of the hyper parameters C and γ optimization problem. For a selection of parameters C and γ, parameter tuning was performed on values C ∈ [2^0^, 2^1^,…, 2^4^] and γ ∈ [2^−8^, 2^−7^, …, 1].

R package *e*1*071* is performed for SVMs implementation. We have repeated simulated and real datasets subjected to 100 repeats of 10-cross-validation throughout the analysis.

## Results

### Simulated data results

We analysis simulated dataset where *n*_*1*_ = *n*_*2*_ = 150; p = 25, 50, 100 for evaluating the performance of rank features during the classification. The classification accuracy of 10% to 50% rank features and all features were evaluated.

**Figure 2:**
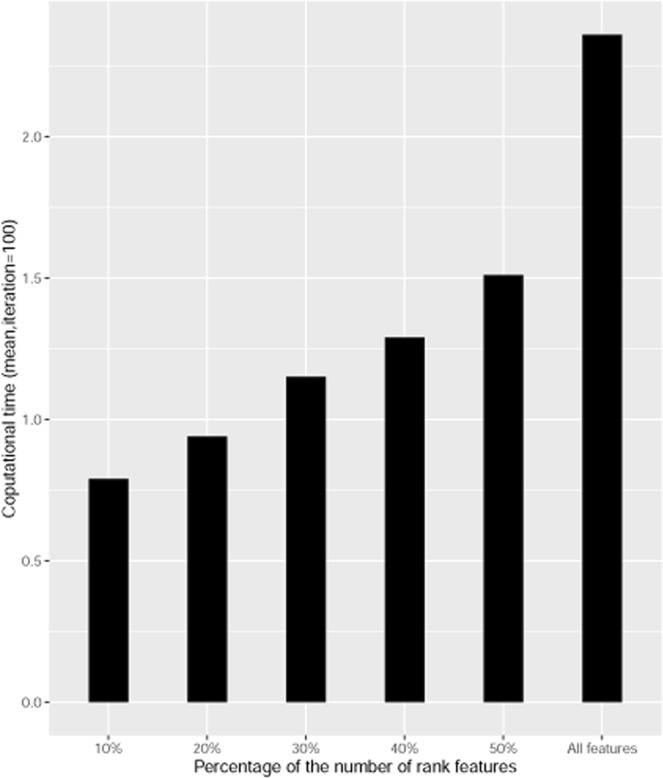
Performance of the number of percentage of the rank features according to the computational time.

When the considered features p = 25, Table 1 shows that the classification accuracy is around 98% for only 10% rank features. We calculated classification accuracy for 20%, 30%, 40%, 50% rank features. All has provided almost same classification accuracy like a non-rank all features. Here, up to 50% rank features have reduced and provided good results (≥98%). The more features means more complexity during training the model, and sometimes it provides misleading results due to the lack of meaningful features in the dataset. Figure 2 is an illustration of the performance of the number of percentages of rank variables based on computational times. It indicates that, as the percentage of the variable increases, the computational time also increases. However, from 10% to 50% rank features based classification model computational time is much lower than that the computational time of the model which contains all the features, but performance is similar.

**Table 1:** Average classification accuracy (%) of the simulated data (p = 25) subjected to 100 repeats of 10-cross-validation based on rank features.

Rank Features Accuracy						
ML Methods	10%	20%	30%	40%	50%	All features (100%)
LDA	98.21	98.87	99.41	99.63	99.86	100.00
RF	97.30	97.56	97.70	97.78	97.81	97.90
SVM-*l*	98.08	98.65	99.07	99.22	99.39	99.53
SVM-*r*	97.88	98.34	98.55	98.61	98.67	98.53

For p = 50, 10% rank features classification accuracy is more than 90%, 20% rank features classification accuracy is around 93%, 30% rank features classification accuracy is 93%, 40% and 50% rank features classification accuracy are almost same as like as without rank features for all ML methods except RF. But RF accuracy is more than 91% (Table 2).

**Table 2:** Average classification accuracy (%) of the simulated data (p = 50) subjected to 100 repeats of 10-cross-validation based on rank features.

Rank Features Accuracy						
ML Methods	10%	20%	30%	40%	50%	All features (100%)
LDA	91.97	93.29	94.32	95.19	95.78	100.00
RF	91.15	91.48	91.66	91.73	91.79	91.91
SVM-*l*	91.82	92.90	93.62	94.23	94.73	95.07
SVM-*r*	91.48	92.62	93.33	93.67	93.75	93.13

When p = 100, all the ML methods prediction accuracy was more than 80% with 10% rank features. We increased the percentage of the rank features, and then prediction accuracy also increased. When we choose rank features up to 50%, LDA and SVM-*l* accuracy are more than 90%. However, when we used all the features during classification, the prediction accuracy was equivalent to the 50% rank features. The classification accuracy of all the ML methods for p = 100 is shown in Table 3. In the simulation study, it was also noticeable that, among the ML algorithms RF prediction accuracy has decreased only when the number of variables in the dataset increased (p = 100). Otherwise, their performance was almost similar in all cases.

**Table 3:** Average classification accuracy (%) of the simulated data (p = 100) subjected to 100 repeats of 10-cross-validation based on rank features.

Rank Features Accuracy						
ML Methods	10%	20%	30%	40%	50%	All features (100%)
LDA	84.97	87.23	89.30	91.10	92.43	94.88
RF	83.55	83.80	83.89	83.92	83.87	83.87
SVM-*l*	84.49	86.53	88.21	89.50	90.39	91.45
SVM-*r*	84.42	86.78	88.03	88.62	89.04	87.21

This classification accuracy we have obtained based on the mean difference of populations (*m*) which was 9 to 10 by 0.01. We have generated simulated data 100 times and taken average corresponding ML methods classification accuracy. Since simulation study proved that up to 50% rank features prediction accuracy is almost same when we are using all the non-rank features. Therefore, we used up to 50% rank features after processing real dataset for plant status detection, and validating/evaluating the performance of the rank features based on prediction accuracy of the ML methods used in this study.

### Real data results

**Figure 3:**
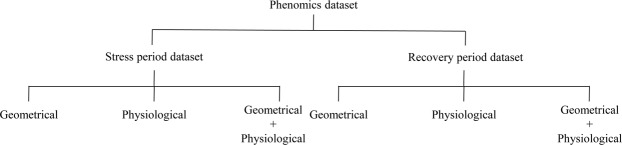
Plant phenotype dataset. Dataset preparation based on features categories of two plant growing period.

We analysis two growing period (stress period and recovery period) plants phenotype datasets, and divided it into six datasets based on phenotypic traits category. The last day of stress and recovery period datasets with geometrical (Geo) and physiological (Phy) traits has been analyzed (Fig. 3). These datasets have processed and obtained meaningful traits by following the first phase of our proposed framework (processing) summarized from Chen *et al*.^9^. Then we ranked (the second phase) ‘geometrical’, ‘physiological’ and ‘geometrical + physiological’ traits, and evaluate the selected traits (features) performance by the prediction of plant status (stress/non-stress).

**Figure 4:**
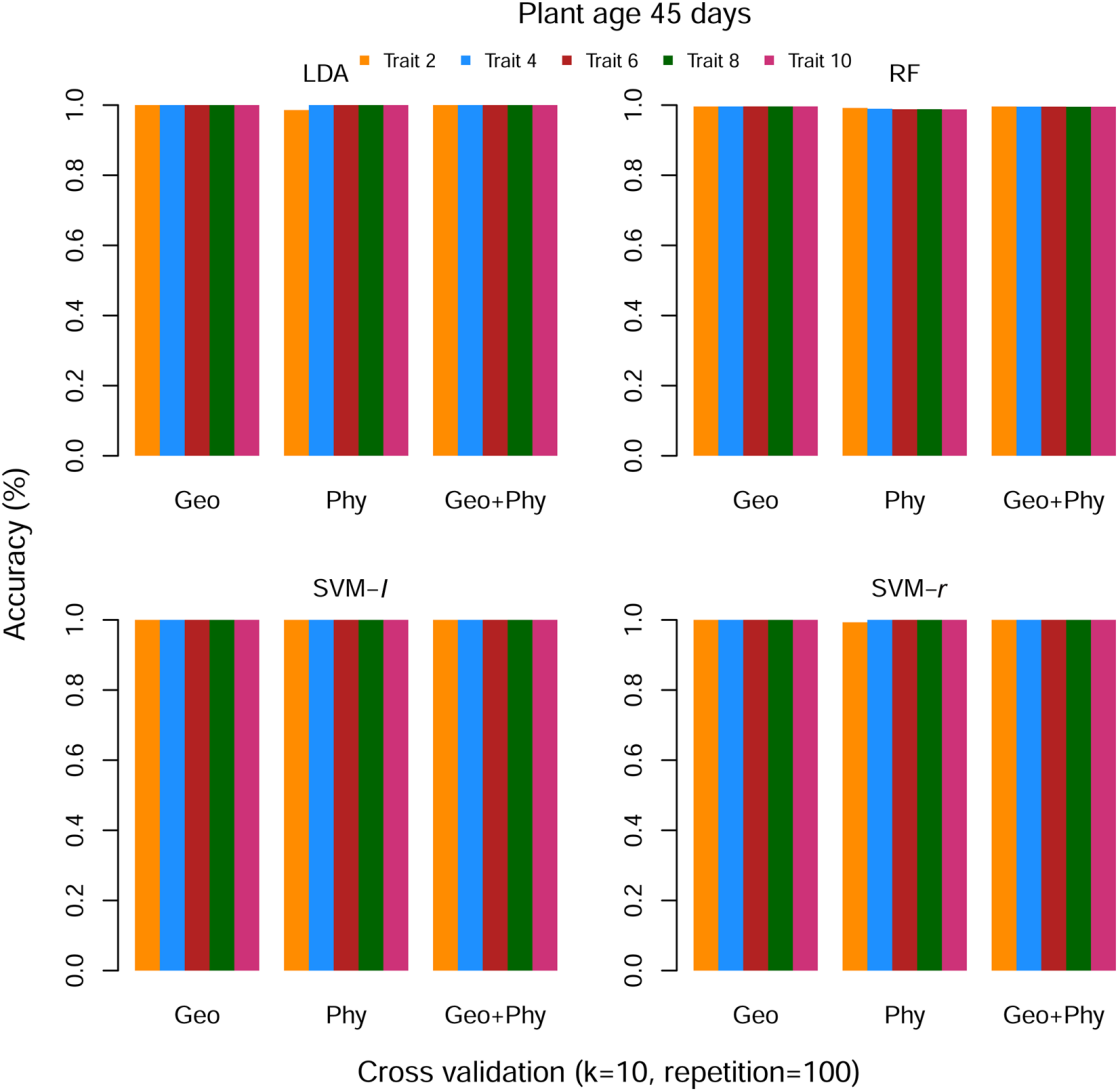
Performance of rank features for stress period data set. ‘Geo’ is geometrical, ‘Phy’ is Physiological and ‘Geo + Phy’ is combined Geometrical and Physiological features.

**Figure 5:**
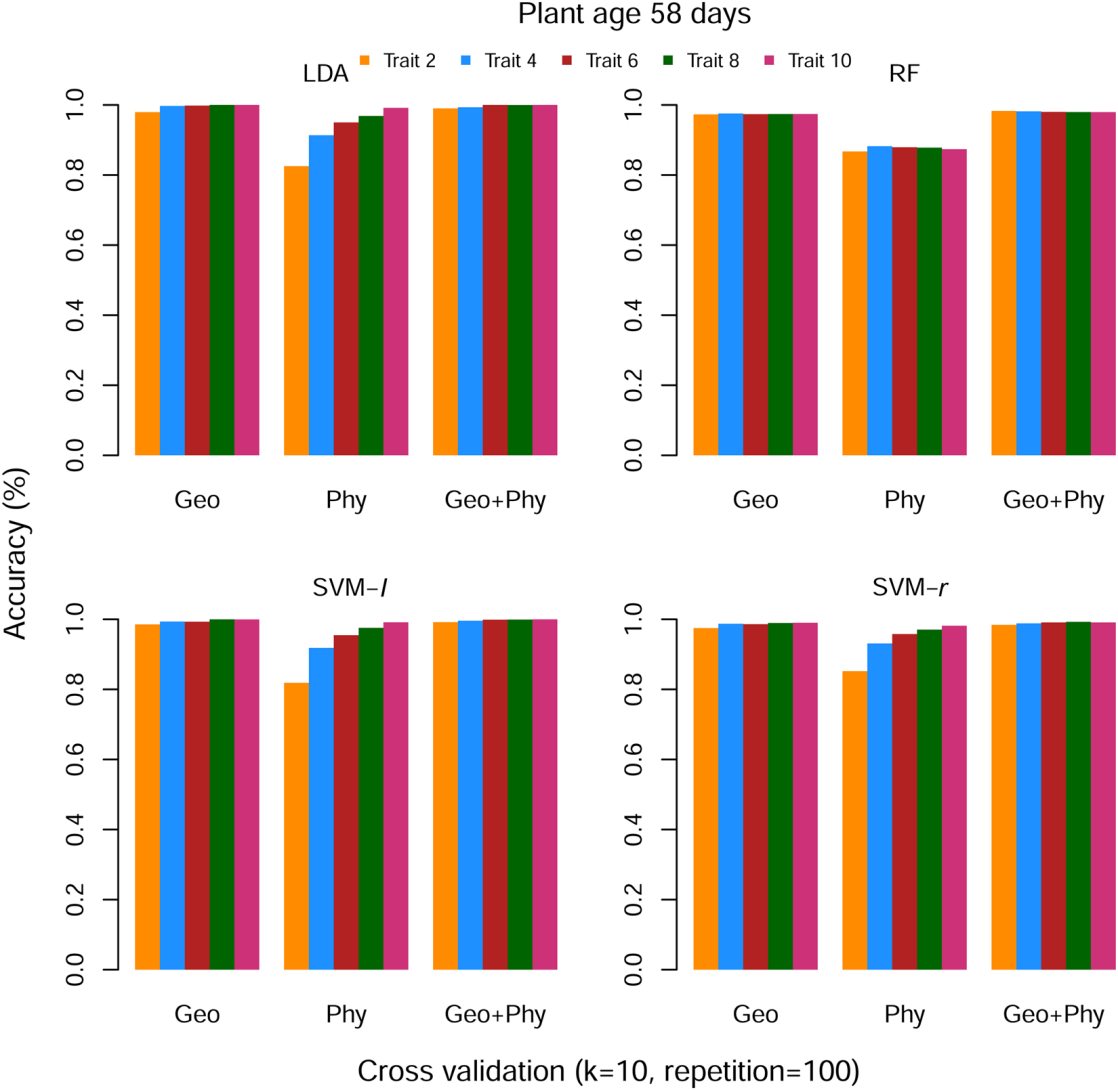
Performance of rank features for recovery period data set. ‘Geo’ is Geometrical, ‘Phy’ is Physiological and ‘Geo + Phy’ is combined Geometrical and Physiological features.

Using *k*-fold cross-validation method, we split the dataset and* k*-1 set data was used for the training model, and rest set of data was used for testing, here *k* = 10. This procedure was repeated 100 times. The obtained results were an average of the classification accuracy for each sets of data. In stress period dataset, only the first two ranked features have provided almost 100% classification accuracy for all categories of features. Then we sequentially added four to ten features and observed that the accuracy has unchanged (Fig. 4). In recovery period data, classification accuracy is 99.99% for Geo (geometrical) rank features, whereas Phy (physiological) rank features classification accuracy is 80% when a number of rank features are 2. After sequentially adding rank Phy features, classification accuracy has improved and when a number of rank Phy features is 10 then the prediction accuracy turn into 100%. Similar accuracy results were found for the SVM-*l* and SVM-*r*, whereas RF has provided lower accuracy (≤85%) for Phy rank features. However, in this dataset, combined Geo and Phy (Geo + Phy) rank features prediction accuracy is 99.98% for all ML methods on average (Fig. 5). The standard error among the accuracy is ≈0.

**Figure 6:**
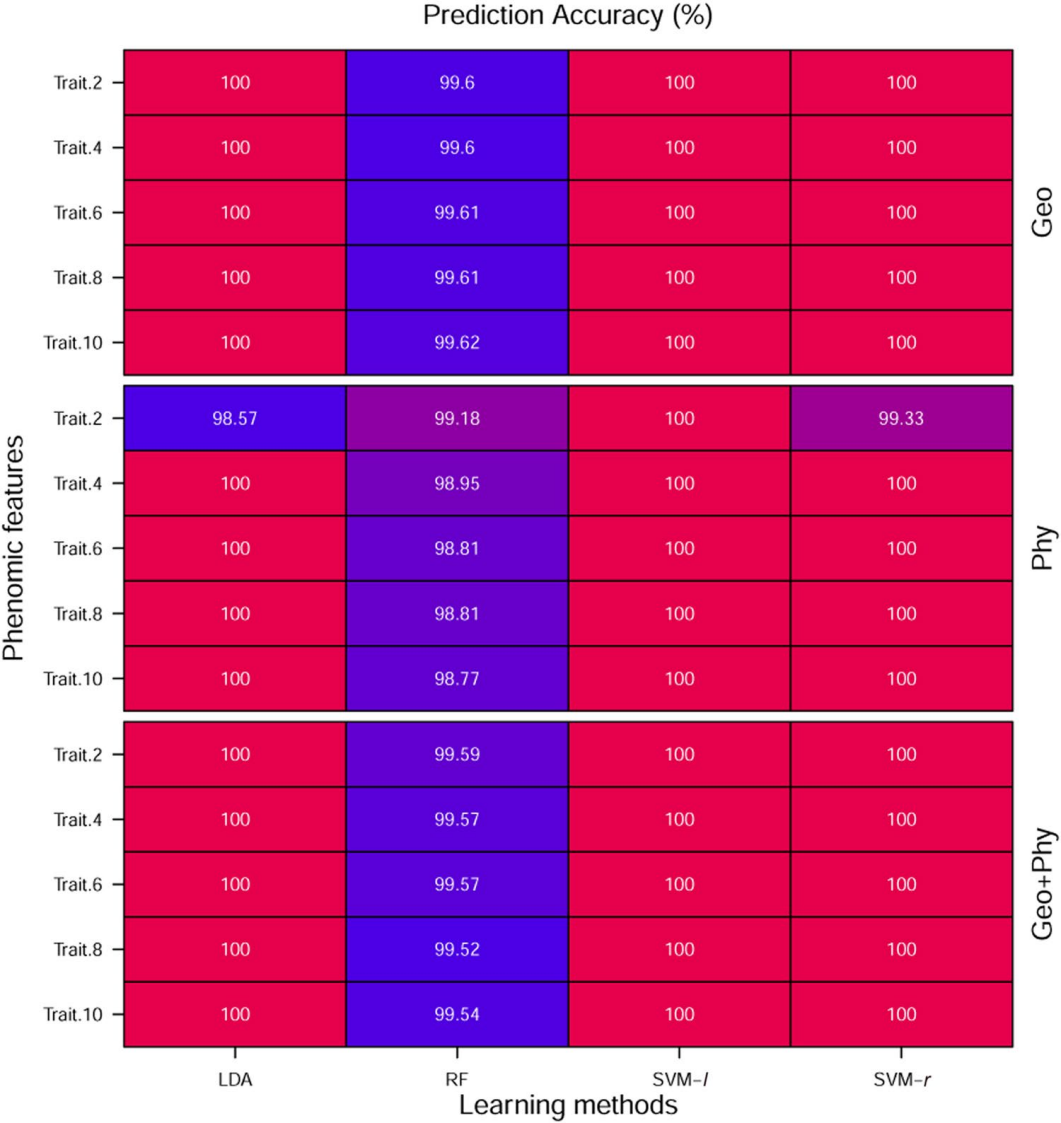
Comparison of classification accuracy of ML methods based on rank features for stress period data set. The Number of rank features is shown on the left and features categories are shown in the right of panels, respectively. In each column of panels, the results from a different type of ML methods are shown. Every ML method was subjected to 100 repeats of 10-cross-validation and the results shown are the average of the classification accuracy. The value in each cell is color coded (0, 1), ranging from red to blue.

**Figure 7:**
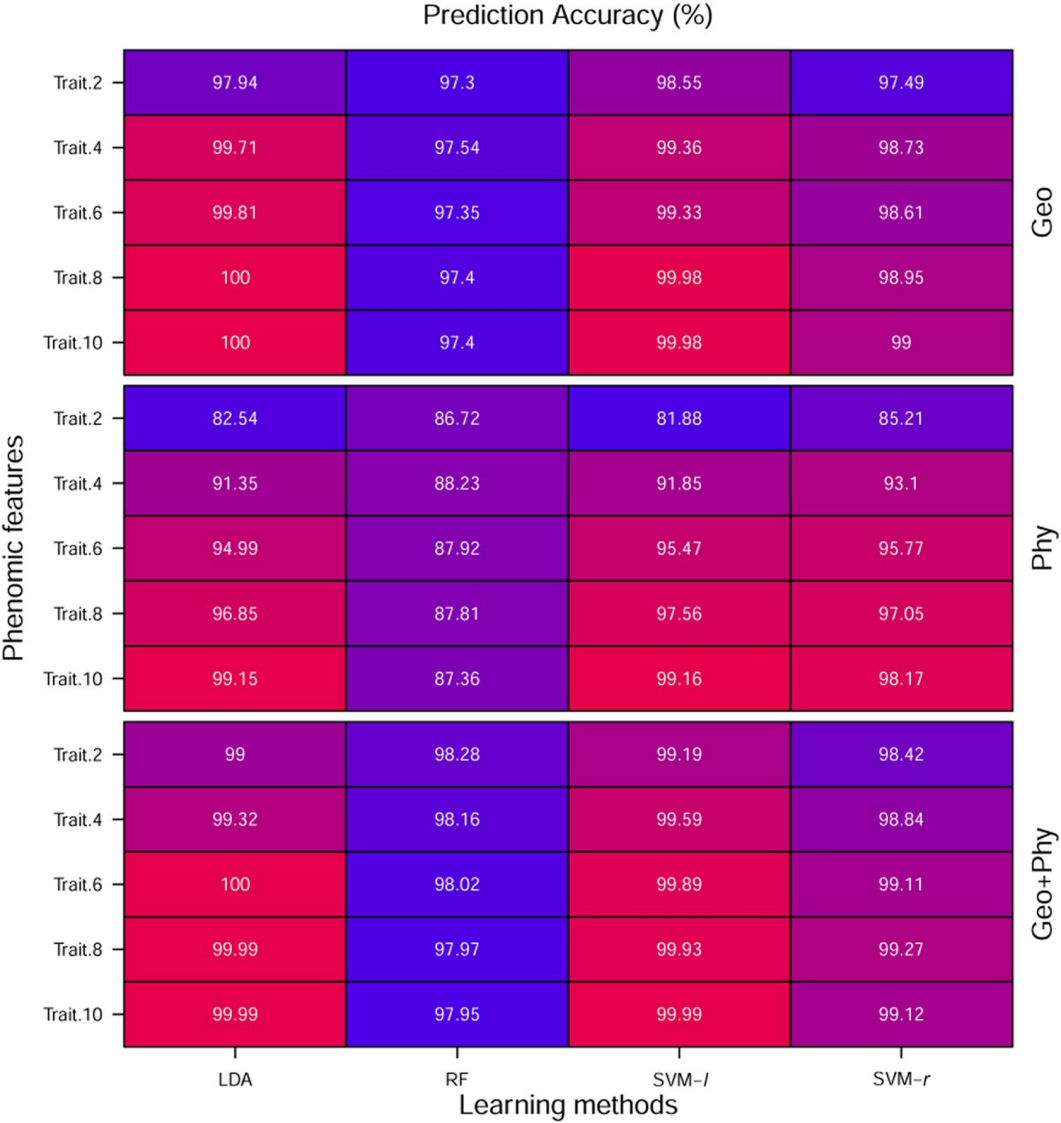
Comparison of classification accuracy of ML methods based on rank features for recovery period data set. The Number of rank features is shown on the left and features categories are shown in the right of panels, respectively. In each column of panels, the results from a different type of ML methods are shown. Every ML method was subjected to 100 repeats of 10-cross-validation and the results shown are the average of the classification accuracy. The value in each cell is color coded (0, 1), ranging from red to blue.

Figures 6 and 7 describe a comparison among the ML methods for both stress and recovery period dataset, respectively. In the case of stress dataset, LDA and SVM-*r* prediction accuracy are 100% for Geo, Phy and Geo + Phy rank features (the number of rank features are 2, 4, 6, 8, and 10) except when Phy rank features are 2. SVM-*l* outperforms than others and its prediction accuracy is 100% for all categories rank features. Although, RF is slightly worse classification accuracy than LDA, SVM-*l* and SVM-*r*; however its prediction accuracy is more than 97% on average. For recovery period data, LDA and SVM-*l* prediction accuracy are 99.99% and 99.15% when the number of rank features are 10 of Geo + Phy and Phy, respectively. Whereas LDA accuracy is 100% when the number of rank features of Geo are 10. RF suffers lower performance and its prediction accuracy of all categories features is more than 97% except Phy features, even though a number of rank features we have taken up to 10. However, there is no noticeable difference in the performance of LDA and SVM-*l* for the recovery period dataset. Overall, all the ML methods in the real data analysis, the classification accuracy reached an acceptable level of performance for all cases throughout the analysis.

## Discussion

DM and ML is an inherently multidisciplinary approach to data analysis that draws inspiration, and borrows heavily, from statistics, probability theory, decision theory, optimization, and visualization. DM and ML methods are typically useful in situations where big data problems are available. Several image-based studies have used and evaluated DM and ML methods performance in biology and images obtained in high-throughput screening^31,34–37^. The enormous volume, variety, velocity, and veracity of imaging and remote-sensing data generated by such real-time platforms represent a ‘big data’ problem.

High-throughput plant phenomics technologies have resulted in an inundation of high-resolution images and sensor data of plants. Extracting these data patterns and features requires powerful statistical approaches for increasing amount of phenotyping information of plants. Combining DM and/or integrating ML methods for plant phenomics data pre-processing, variable selection and group classification, respectively, might overcome this big data analysis problem^4^. One of the major benefits of using DM and ML approaches for plant breeders, physiologists, pathologists, and biologists is the opportunity to search large data sets to discover patterns and govern discovery by simultaneously looking at a combination of factors instead of analyzing each feature (trait) individually. Previously, this was a major bottleneck because the high dimensionality of individual images makes them extremely hard to analyze through conventional techniques. Another key challenge that the underlying processes for linking the inputs to the outputs are too complex to derive mathematical models^3^.

Previous studies have applied ML methods for feature selection, feature ranking and classification based on root features of phenomics data^4,7,9^. Integrated methods or powerful techniques improved the accuracy of the data analysis confirming earlier results by Löw *et al*.^38^ and Zhao *et al*.^4^. We combined DM and ML methods for feature selection, feature ranking and classification, and the performance accuracy is much better (≥98%) for all the classifiers on an average.

We used shoot image features in this study. Our results clearly demonstrated the importance of selecting important features to obtain efficient classification results for the phenomics dataset. The improved accuracy probably benefits from alleviating the ‘curse of dimensionality’ through rank features selection by removing less informative features during classification. The ‘Geo’ features are the most important features performing better than ‘Phy’ feature in case of recovery period data for all ML methods. Although, ‘Phy’ features performing same as like as ‘Geo’ features in case of stress data set. The combined ‘Geo’ and ‘Phy’ feature performing well in both cases of the datasets. The classification performance of ML methods increases when rank features not more than 50%. The overall prediction accuracy of the ML methods was cross-validated.

In summary, our study advocates that among the considered ML methods except RF, there is no noticeable difference among the classification accuracy, when the features was selected through our proposed approach. This approach reduces the computational time as well as increases the classification accuracy power by adding rank features sequentially for achieving acceptable performance of the algorithms. However, LDA is good when data are normally distributed and there is no curse of dimensionality, otherwise it does provide misleading results. RF accuracy is much lower than other ML methods for both the simulated and real dataset used in this study. SVMs are the appropriate choice for high-throughput phenomics data analysis (especially SVM-*l* in the iterative training of the classifier to classify all the phenotype data including classifying unlabeled plant phenotype dataset).

## Conclusions

The accurate classification of stress plant (accuracy more than 98% on average) indicates that rank features performed well which were selected through our proposed approach. In particular, this study showed that the combined DM and ML method for trait identification and classification, respectively, can overcome problems in applying ML approaches to analysis phenotype data. Hence, the proposed approach is generally useful to make plant phenotype data analysis more effective and robust throughout the classification. We conclude that this proposed analytical approach, in advance our views can be useful for image-based plant phenotype data processing and finding complex traits for the study of QTL (Quantitative Trait Locus) or GWAS (Genome-wide Association Study), stress identification, disease prediction, and for further statistical investigation of phenomics dataset in plant growth and development research.

## Data Availability

The phenotype image data we downloaded from http://iapg2p.sourceforge.net/modeling/#dataset, and the R code is available upon request.
